# Judgment of the Humanness of an Interlocutor Is in the Eye of the Beholder

**DOI:** 10.1371/journal.pone.0025085

**Published:** 2011-09-22

**Authors:** Catherine L. Lortie, Matthieu J. Guitton

**Affiliations:** 1 Centre de Recherche Université Laval Robert-Giffard (CRULRG), Quebec City, Quebec, Canada; 2 Faculty of Pharmacy, Laval University, Quebec City, Quebec, Canada; Hungarian Academy of Sciences, Hungary

## Abstract

Despite tremendous advances in artificial language synthesis, no machine has so far succeeded in deceiving a human. Most research focused on analyzing the behavior of “good” machine. We here choose an opposite strategy, by analyzing the behavior of “bad” humans, i.e., humans perceived as machine. The Loebner Prize in Artificial Intelligence features humans and artificial agents trying to convince judges on their humanness via computer-mediated communication. Using this setting as a model, we investigated here whether the linguistic behavior of human subjects perceived as non-human would enable us to identify some of the core parameters involved in the judgment of an agents' humanness. We analyzed descriptive and semantic aspects of dialogues in which subjects succeeded or failed to convince judges of their humanness. Using cognitive and emotional dimensions in a global behavioral characterization, we demonstrate important differences in the patterns of behavioral expressiveness of the judges whether they perceived their interlocutor as being human or machine. Furthermore, the indicators of interest displayed by the judges were predictive of the final judgment of humanness. Thus, we show that the judgment of an interlocutor's humanness during a social interaction depends not only on his behavior, but also on the judge himself. Our results thus demonstrate that the judgment of humanness is in the eye of the beholder.

## Introduction

As the use of the Internet and virtual reality applications is largely spreading into everyday situations, the need for human-like autonomous agents is rapidly growing [1], [2]. One of the main criterions in creating a convincing human-like autonomous agent is its ability to imitate a human in a persuasive way [3]–[7]. The extending use of virtual agents in medical and educational fields, such as in phobia treatment or physical rehabilitation, imposes the humanization of artificial agents as one of the top priorities for applied cognitive sciences in the near future.

Technologies supporting the design of virtual spaces have now reached a level of maturity advanced enough to obtain highly convincing results regarding purely visual aspects of agents [8]. Since decades, numerous authors have improved artificial agents by studying and modulating specific aspects important in human interactions, such as physical appearance [8], [9], body shape [10], [11], movements [12], [13], or voice [14], [15].

However, attempts to convince subjects of the humanness of an artificial agent have remained unsuccessful [16]. Indeed, physical factors are not the only elements that have to be taken into account. Particularly, the behavior of an agent is a central component of its human-likeliness. In other words, human-like agents should be able to display advanced cognitive abilities (e.g., social skills, intelligence, language) in order to be credible as a communication partner [1], [4], [6]. This goal has not been achieved yet, as present agents are still not cognitively convincing in a way that leads human subjects to believe that they are interacting with a real human being.

Since the early days of artificial intelligence research, language has been identified as the main output to test the human-likeliness of artificial agents' cognitive abilities in experimental settings. The pioneer of the artificial intelligence field, Alan Turing, proposed a now famous test in order to determine the capability of an agent to mimic humans [6]. The Turing Test is an experimental situation in which a subject and a computerized program, hidden behind a screen, communicate with a human examiner through text messages. If the examiner is unable to determine which terminal is controlled by a human subject and which is controlled by a computerized program, the latter is said to pass the Turing Test, i.e. the computer is indistinguishable from a human subject.

The efforts of robotics or computer-mediated communication researchers have yet to be successful in creating an agent able to pass the Turing Test [4], [16]. Confronted with this challenge, we were interested in taking a different approach at this “false human” agent problem. Since previous unsuccessful attempts focused primarily on improving agents' cognitive – and more important linguistic – credibility, it became obvious that another point of view was needed in order to improve actual comprehension of interactions between artificial agents and real humans.

At the current state of knowledge, what the best software can teach us is still limited, due to inherent biases which have been led by the programming. Since the earliest work in computerized language, the vast majority of researchers studied the linguistic and interaction behavior of the best agents available [4], [17]–[19]. We here decided to undertake a Copernican revolution. Instead of focusing on “good robots”, we focused on “bad humans”, i.e., on humans who have been perceived as non-humans by independent judges. We thus asked ourselves if it would be possible to identify the exact parameters of a successful interaction by analyzing the linguistic behavior of subjects judged as machines in a computer-mediated communication task.

To answer this crucial question, we selected as a model the Loebner Prize in Artificial Intelligence. The Loebner Prize is a recent version of the Turing Test, using linguistic production in the form of dialogue between programs, human subjects, and judges. Since 1991, this yearly contest gathers human participants and machines (linguistic software) that try to convince judges about their humanness by communicating through computer terminals without seeing each other. After the conversations, the judges decide which terminals were controlled by humans and which ones were controlled by programs.

However, up to now, a highly interesting aspect of the Loebner Prize has been neglected. Throughout the years, some human participants have been perceived as *machines* by the judges. While programs attempt to fool the judges so they believe in their humanness, human subjects should not have to make any particular effort to convince other humans that they are indeed who they claim to be. What could have happened during the interaction so that human beings were considered as non-humans? What parameters need to be present to guarantee a positive identification of a human within a social interaction narrowed down to computer-mediated communication?

Instead of studying good robots, this new approach of examining the linguistic behavior of “bad” human participants focused on how human beings could have been considered as robots during the course of a blinded social interaction. The Loebner Prize model enabled us to study semantic aspects of a dialogue that can deceive a judge on his interlocutor humanness, by examining which faulty events have created such a negative behavioral response toward the participant. Using cognitive and emotional dimensions in a global behavioral characterization, we suggest the importance of multimodal, emotional and cognitive parameters when analyzing complex social behaviors. Our results demonstrate that the judgment of an interlocutor' humanness during a social interaction not only depends on his behavior, but also on the behavior of the judge himself. Thus, we demonstrate that the judgment of humanness is in the eye of the beholder.

## Methods

### 1. Experimental model

Dialogues were extracted from the *Loebner Prize in Artificial Intelligence*. During this annual contest, subjects and conversational programs try to convince judges of their human nature through computer-mediated communication. No conversational program has yet succeeded in fooling judges, but some human subjects failed to convince the judges of their human nature and were judged as robots by at least one of them. In the present study, we focused on the characteristics of the dialogues between human subjects who have been judged at least once as robots and their respective judges. The linguistic productions were thus divided into four groups: subjects judged as humans, the same subjects judged as robots, judges making a human judgment of their interlocutor, and judges making a robot judgment of their interlocutor.

### 2. Data collection

Dialogue samples were collected via transcriptions available on the *Loebner Prize in Artificial Intelligence* website database (http://www.loebner.net/Prizef/loebner-prize.html). Dialogues in which subjects were considered as robots by a minimum of one judge were selected. Over the years of the Loebner Prize, several ranking techniques were used to determine which contestants seemed more human than others. When judges were asked to separate the terminals that they believe were controlled by humans of those that they believed were controlled by machines (e.g., year 1992 or 2010), their judgment indicated which participants were considered as robots. When judges were asked to give each terminal a “humanness” score between “definitely a machine” to “definitely a human” on a five points Likert scale, we considered that the followings judgments “definitely a machine” and “probably a machine” meant the judges considered their interlocutor to be a robot (e.g., year 2003). When judges were asked to rank terminals between 1 as being a human to 6 as being a robot, we considered that the followings ranks 4, 5 and 6 meant the judges considered their interlocutor to be a robot (e.g., year 1997 or 2009). Finally, when judges were asked to divide a percentage between 2 terminals according to their humanness (100% meaning being a human without any doubt – for instance, if a terminal get 90%, the other one would get 10%), we decided that 60% was the breaking point from which a terminal would be considered as a robot (e.g., year 2004 or 2005).

Analyses could only be performed when the logs of all subjects' conversations and their detailed ratings were available. The dialogue transcripts of the subjects and their respective judges were used for further analyses. All transcripts were first saved in a text format compatible with Microsoft Word software and then normalized in order to carry out linguistic analyses.

### 3. Data analysis

The parameters used to analyze the dialogues were gathered in three broad categories: descriptive parameters, cognitive parameters and indicators of interest. Descriptive parameters were: number of words, sentences, posts, mistakes, words per sentence, words per post, sentences per post, number of social words, long words (more than 6 letters), positive emotion words, negative emotions words, total emotions words, articles (a, an, the), greetings at beginning, greetings at end, and acknowledgments. Cognitive parameters were: number of self-references (I, me, my), references to relatives (family and friends), compliments, occurrence of aggressiveness, and occurrence of emotions (fear, happiness, angriness, surprise or disgust). Indicators of interest were: number of questions, questions per post, and overall number of cognitive words used.

Parameters were collected using classification grids or the Linguistic Inquiry and Word Count program (LIWC) [20]. LIWC is a text analysis software program which uses an internal dictionary to categorize words of a text file, and then calculate a percentage of occurrences for each word categories used in the text, as the number of words in a given category divided by the text's total length. LIWC' validity of measure has been demonstrated for emotional expression presented in text [21] and for detecting attention focus, thinking style, emotionality, social relationships, and individual differences [22]. Furthermore, LIWC has been used to examine text samples in online format in many studies (e.g., [23], [24]).

Patterns of behavioral expressiveness were built using five dimensions selected accordingly to their relevance for inter-individual interactions. The five dimensions selected were occurrence of aggressiveness, self-references, references to relatives, compliments and occurrence of emotions. The data for each of the four groups were then normalized depending on their relative importance across the groups.

### 4. Statistical analysis

Analysis of the different parameters was performed using the non-parametric Wilcoxon paired test or Student paired t-tests, when the normality of the distribution allowed it. Comparisons were made between two groups (subjects judged as human vs. subjects judged as robots; subjects judged as human vs. judge judging as human; subjects judged as robots vs. judge judging as robots; judge judging as human vs. judge judging as robots). Patterns of behavioral expressiveness were compared using the non-parametric distribution free Kolmogorov-Smirnov analysis of the parameters' distributions, in order to account for differences in the patterns of behavioral expressiveness between the groups. When applicable, results are presented as mean ± SEM.

## Results

The main findings of our study were the evidence of important differences in the patterns of behavioral expressiveness of the judges whether they perceived their interlocutor as being human or machine. Subjects judged as robots used fewer words per post, fewer long words and fewer articles than those judged as humans. Furthermore, subjects judged as human made more posts and more compliments than did the judges perceived them as been human. They also used more words per post, more long words and more articles than the judges judging as human. Finally, judges judging as humans asked more questions, more questions per post and used more cognitive words than did subjects judged as human.

### 1. Sample characteristics

All transcripts available since the first year of the Loebner contest in 1991 were collected. Data were available for years 1992, 1995, 1996, 1997, 1998, 1999, 2000, 2001, 2003, 2004, 2005, 2006, 2007, 2008, 2009, and 2010. However, several years of the contest had to be excluded from the sampling. Specifically, detailed ratings of the dialogues were not available for 5 years of the contest (1995, 1996, 2006, 2007, and 2008), and the dialogues of 2001 contest were not available. During 6 of the remaining years of the contest (years 1997, 1998, 1999, 2000, 2009 and 2010), no subject was considered as a machine. Our final sampling thus consisted of 4 years of the Loebner Prize (years 1992, 2003, 2004, and 2005).

Among these 4 years, a total of 9 subjects were judged at least one time as a machine by at least one judge (Table 1). These subjects generated 57 dialogues with their corresponding judges (6.33±.83 dialogues per subject), for an overall total of 21,780 words (Table 1). Among those 57 dialogues, 16 (28.1%) were rated by the judges as being produced by a machine, and 41 (71.9%) as being produced by a human.

**Table 1:** Description of the sample.

Subject	Year	Judged as human	Judged as robot	Total conversations	Total words
1	1992	6	2	8	2,965
2	1992	6	2	8	3,119
3	1992	5	3	8	3,529
4	2003	6	3	9	2,492
5	2003	7	2	9	2,299
6	2004	2	1	3	2,794
7	2005	3	1	4	1,294
8	2005	3	1	4	1,443
9	2005	3	1	4	1,845
	Total	41	16	57	21,780

For each subject judged at least once as a robot across the years of the Loebner Prize, the columns indicate the number of times the subject was perceived as a human or as a robot by the judges, the year corresponding to the occurrence of the contest, and the total number of words spoken both by the subject and the judges during the conversations.

### 2. Descriptive parameters

Significant differences were observed for several structural parameters. In particular, in the number of posts (*p*<.05 between the subjects judged as human and the judges judging as human, Table 2) and the number of words per post (*p*<.05 between the subjects judged as human and the subjects judged as machine, Table 2). In addition, some non-significant trends were also observed in other structural parameters, such as the total number of words, the number of sentences, the number of words per sentence, and the number of sentences per post (Table 2).

**Table 2:** Descriptive parameters.

	Humans judged as human	Humans judged as robot	Judges judging as human	Judges judging as robot	Significance
*Structural parameters*					
Number of words	244,24±47,91	244,89±60,62	178,76±16,79	174,82±27,79	
Number of sentences	27,05±2,94	27,96±4,78	23,76±2,61	22,43±3,0	
Number of posts	**13,58±1,30**	15,26±1,81	**12,94±1,19**	14,82±1,80	p < .05 (W)
Number of mistakes	11,19±4,70	19,74±12,14	9,42±1,34	18,54±5,38	
Number of words per sentence	9,30±1,31	8,58±1,17	7,86±0,56	7,80±0,63	
Number of words per posts	**20,64±4,37**	**16,77±3,18**	14,66±1,37	12,70±1,67	p < .05 (T)
Number of sentences per posts	2,11±0,17	1,82±0,14	1,88±0,10	1,59±0,14	
Number of articles	**7,69±0,56**	5,69±0,52	**6,21±0,35**	5,58±0,80	p < .05 (T)
	**7,69±0,56**	**5,69±0,52**	6,21±0,35	5,58±0,80	p < .05 (T)
*Linguistic parameters*					
Number of social words	10,09±0,97	11,15±1,37	10,30±0,58	10,26±1,31	
Number of long words	**14,76±1,29**	13,48±1,30	**12,06±0,51**	12,29±1,24	p < .05 (T)
	**14,76±1,29**	**13,48±1,30**	12,06±0,51	12,29±1,24	p < .05 (T)
Number of positive emotions words	3,42±0,40	3,35±0,34	3,38±0,27	3,02±0,49	
Number of negative emotions words	1,17±0,18	1,35±0,27	0,93±0,16	0,99±0,25	
Number of total emotions words	4,59±0,44	4,70±0,37	4,32±0,36	4,01±0,69	
Number of greetings at beginning	60,67±8,92	46,22±14,65	76,33±12,22	66,67±16,67	
Number of greetings at end	29,89±12,81	20,33±11,71	28±13,19	33,33±16,67	
Number of acknoledgments	32,89±8,73	53,67±15,66	35,11±11,49	53,67±15,66	

Analysis of the descriptive parameters of the dialogues for each of the four groups. Results are presented as mean ± SEM. Significant differences are indicated in **bold**. Statistical analyses were performed using Wilcoxon paired test (W) and Student paired t-test (T).

Statistical differences were also assessed in linguistic parameters such as the number of long words (words of more than 6 letters; *p<*.05 between the subjects judged as human and the subjects judged as machine, and *p*<.05 between the subjects judged as human and the judges judging as human, Table 2) and the number of articles (*p*<.05 between the subjects judged as human and the subjects judged as machine, *p*<.05 between the subjects judged as human and the judges judging as human, Table 2). However, no significant difference was found between the groups on positive emotion words, negative emotion words and total emotion words.

Surprisingly, no significant difference was observed in the number of social words or the number of mistakes (Table 2). Finally, no significant difference was assessed between the four groups on the expression of indicators of courtesy, i.e., in the number of greetings at the beginning of the dialogues, the number of greetings at the end of the dialogues, nor the acknowledgements (Table 2).

### 3. Cognitive parameters

**Figure 1:**
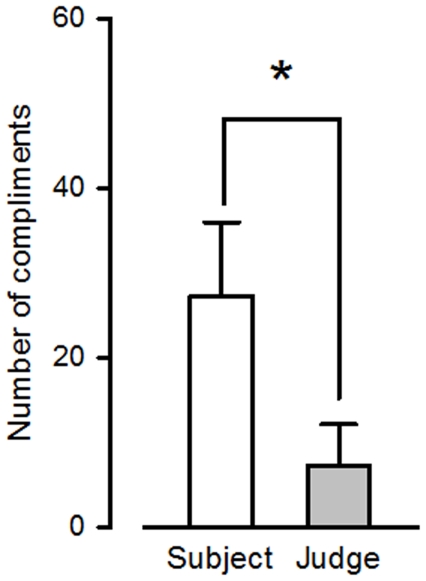
Number of compliments. Number of compliments that the subjects and the judges gave each other when the subjects were perceived as humans, *p*<.05 (Student paired t-test).

Cognitive parameters were observed both independently and in combination as patterns of behavioral expressiveness. When taken individually, almost no effect was assessed in the different cognitive parameters. A significant difference was evidenced in the number of compliments (*p*<.05 between the subjects judged as human and the judges judging as human, Figure 1), and a trend was also observed in the references to relatives (with subjects judged as robots displaying seemingly more references to relatives than subjects judged as human). No effects were seen in the number of self-references, occurrence of aggressiveness, and occurrence of emotions (fear, happiness, angriness, surprise or disgust).

### 4. Patterns of behavioral expressiveness

**Figure 2:**
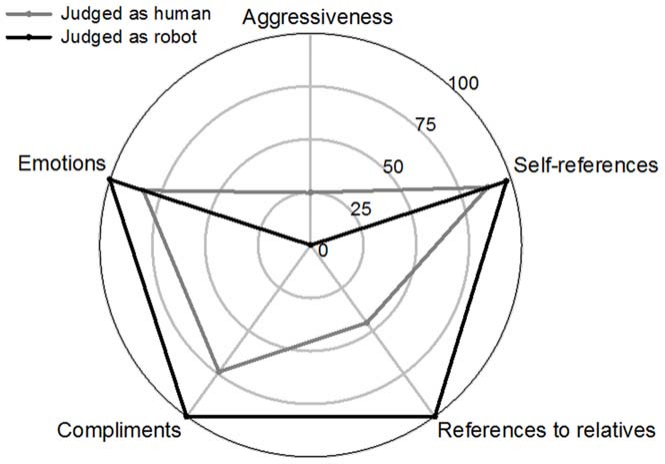
Patterns of behavioral expressiveness of the subjects whether they were perceived as humans or as robots. Patterns are based on the subjects' results on the five dimensions selected (aggressiveness, self-references, references to relatives, compliments, emotions).

**Figure 3:**
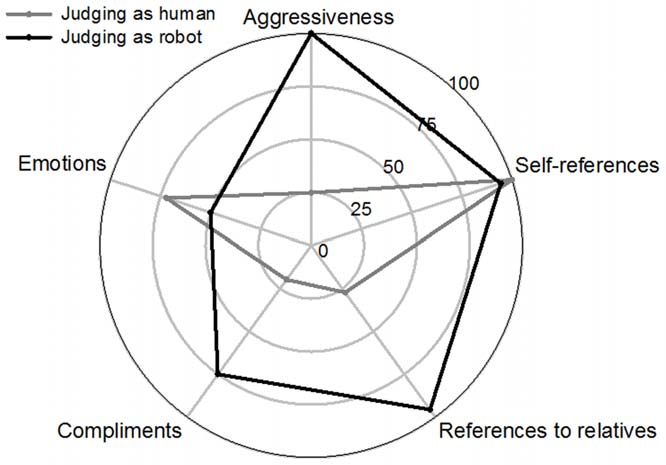
Patterns of behavioral expressiveness of the judges whether they perceived their communication partners as humans or as robots. Patterns are based on the judges' results on the five dimensions selected (aggressiveness, self-references, references to relatives, compliments, emotions). Note that the two patterns are significantly different (*p*<.05, Kolmogorov-Smirnov test).

The patterns of behavioral expressiveness of the subjects did not significantly differ whether they were considered as human or as robot, even if humans perceived as robots tend to display more behavioral expressiveness than when perceived as human, except for aggressiveness (Figure 2). However, a significant difference was observed between the judges' pattern of behavioral expressiveness depending if they perceived their interlocutor as being human or robot, *p*<.05 (Kolmogorov-Smirnov, Figure 3). While the pattern of behavioral expressiveness of the judges judging their interlocutor as human is very similar to the pattern of behavioral expressiveness of the subject, the pattern of behavioral expressiveness of the judges judging their interlocutor as robot was highly different from the one of the subjects, presenting a high level of aggressiveness.

### 5. Indicators of interest

**Figure 4:**
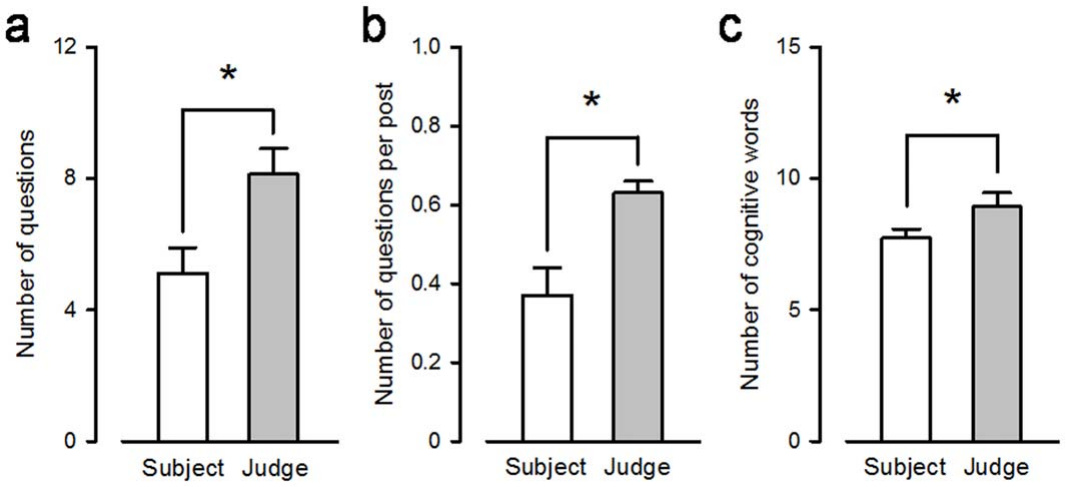
Indicators of interest. (a) The number of questions, (b) questions per post and (c) cognitive words the subjects and the judges have displayed when the subjects were perceived as humans, *p*<.05 (Student paired t-tests).

When the subjects were judged as human, judges displayed significantly more indicators of interest toward the subjects (Figure 4). Differences were assessed in the number of questions (*p*<.05 between the subjects judged as human and the judges judging as human), the number of questions per post (*p*<.05 between the subjects judged as human and the judges judging as human), and the overall number of cognitive words used (*p*<.05 between the subjects judged as human and the judges judging as human).

## Discussion

In the present study, we investigated a large corpus of linguistic interactions in a controlled setting. Our innovative approach allowed us to demonstrate important differences in the patterns of behavioral expressiveness of the judges whether they perceived their interlocutor as being human or machine. In order to be perceived as human, robots should maintain a balanced response to their human interlocutor within each behavioral dimension. Furthermore, the indicators of interest displayed by the judges were predictive of the final judgment of humanness, suggesting that, at least to some extent, the judgment of humanness lies in the eye of the judge himself.

While most researchers investigate the blurred area between perception of humanness and non-humanness – referred as the “Uncanny Valley” in the field of robotics and animation [8], [25], [26] – by improving artificial agents, we tackled the problem at its opposite, i.e., the humans not perceived as such. This new methodological perspective for assessing how to improve the humanness of synthetic agents may be a very useful tactic to investigate other aspects of synthetic agents, such as gesture, facial expression, or more complex behavioural phenomena. Thus, even if the present study focused on some language-related aspects, a similar approach could easily be implemented to study the perception of human-likeness. Ultimately, data generated following this type of approach may have an important impact on future research in the areas of robotics and animation, by providing key factors to designers creating synthetic agents.

### 1. Descriptive parameters

Due to the nature of the Loebner Prize, the fact that the subjects would talk more than the judges was expected. Length of statements is considered as crucial for reciprocal communication [27], [28]. Our results demonstrate that, in a virtual interaction, talking more (e.g., number of posts and words per post) is interpreted positively, as reflected by the judgment of humanness made by the other communication partner. Previous studies have shown that the number of words in linguistic messages has a clear effect on the perception of immediacy [29] and on reactions of self-disclosure by the communication partners [30], both effects obviously contributing to a positive judgment of humanness.

In this setting, subjects who used more articles and more sophisticated words in an overall lengthy dialogue tended to be evaluated more positively – meaning more human-like – by the judges. Accordingly, previous studies have demonstrated that large lexical diversity in speech was usually positively evaluated [31]. In addition, language complexity in computer-mediated communication has been related with immediacy, and perceived as an indicator of care [29]. Some authors also suggested that technical language violations (e.g., spelling and grammatical errors) can have a negative effect on computer-mediated communication [28], [32]–[34]. In the present sample, the presence of spelling or grammatical mistakes did not seem to have an effect on the perception of humanness. However, the overall grammatical quality of the interaction (e.g., well constructed sentences, use of sophisticated words) was clearly associated with a positive judgment of humanness.

### 2. Patterns of behavioral expressiveness

When taken individually, very few of the cognitive parameters analyzed here were significantly associated with a positive judgment of humanness. However, grouping the main cognitive and emotional dimensions in order to define global patterns of behavioral expressiveness pointed to a more complex picture. First, slight differences were assessed between the subjects whether they were judged as human vs. as machine. More importantly, this analysis strategy unveiled significant differences between the expressed behaviors of the judges whether they perceived the subject as human or as machine.

A dialogue is usually characterized by collaborative interactions between the agents. Theoretical models endorse the notion that dialogue coherence is supported by cooperation and balance among agents at each step of the interaction [35]. Without being aware of doing so in social situations, people tend to mimic others laughter [36] and verbal behavior [37]–[39], in a mutual adaptation of linguistic, prosodic, and nonverbal features (for a review, see [40]). Classically, researchers consider only descriptive aspects (such as number of sentences or number of words) to quantify equilibrium [28], [30]. The patterns of behavioral expressiveness observed in this study demonstrate that a break of equilibrium between the communication partners can also be assessed in cognitive and emotional dimensions, and moreover can induce a feeling of unease strong enough to cause a judgment of non-humanness.

When containing only necessary information or lacking conversational tone, computer-mediated communication can be interpreted as rude, and consequently affects perception of likability and friendliness of the interlocutor [32]. A message uncommonly short and deficient in conversational tone causes a communication partner to be seen as lacking of agreeableness, extraversion (i.e., referring to person's sociability) and competent interpersonal skills (i.e., untrustworthiness due to lack of reliability, responsibility and competence) [28]. We demonstrated here that the opposite is also true: a message displaying over-expressivity (as assessed in the patterns of behavioral expressiveness of subjects judged as robots) can have a negative effect on the outcome, i.e. the humanness judgment.

Our results show that self-disclosure (quantified in the present study by the two following parameters: self-references and references to relatives) was not directly associated with the judgment of humanness. However, this broader dimension clearly was one of the main factors responsible for the variability in the patterns of behavioral expressiveness. A balanced self-disclosure displayed between the partners about themselves and their relatives seemed to be associated with a positive response, while too much self-disclosure from an interlocutor led to a disproportional aggressive response. Although not statistically significant, we also observed a tendency to consider as a robot a partner that overused emoticons (e.g. smileys, winks) or excessive laugh. These results are in line with some previous studies. For instance, reciprocity in self-disclosure, as calculated by a positive correlation between the amounts of self-disclosure from the partners, seems to be a significant aspect of interactions [30]. Similarly, it has been shown that a partner could break communication by selecting inappropriate words (such as informal or over-friendly words) in a computer-mediated communication situation [28].

It is important to mention that demographic information of the participants whom behavior was analysed here was not available. Thus, in the present study, it was impossible to decipher whether the age or the gender of the participants may have impacted the perception of humanness. However, previous studies seem to indicate that gender has no effect on reciprocal communication in computer-mediated communication [27], nor on the level of self-disclosure [30].

A key issue is to understand whether the variations of the patterns of behavior expressiveness observed originated from the judge himself, or were triggered by the behavior of the subject aiming to convince the judge of his humanness. If both mechanisms are involved, some of our results suggest that the judge's behavior impacts the evolution of the dialogue and thus the resulting judgment of humanness. Therefore, the quantification of the indicators of interest displayed by the judge – such as direct questioning – is of major importance.

### 3. Indicators of interest

Direct questioning is known to stimulate interpersonal attraction [41], and thus represents an important indicator of interest from a partner during an interaction. Our results show that the judge's questioning was clearly associated with a positive judgment of humanness of the subject. In other words, when a judge was asking more questions – whatever the answers would be –, he was more prone to rate his interlocutor as human. However, the quality of the interlocutor's answers probably conditions the continuity of the judge's interest-based communication strategy.

If indicators of interest are central for interpersonal communication, their form may however vary depending of the experimental situation. Because the Loebner Prize setting favored the linguistic aspect of communication, it may have reinforced the impact of questioning over other indicators of interest.

### 4. Conclusion

In conclusion, by focusing on the linguistic and meta-linguistic behavior of subjects judged as non-human in a computer-mediated communication situation, our results shed a new light on the mechanisms of perception of humanness. We demonstrated here striking differences in the patterns of behavioral expressiveness of the judges whether they perceived their interlocutor as being human or machine. Furthermore, the indicators of interest displayed by the judges were predictive of the final judgment of humanness. These results provide us with a better understanding of the general phenomena underlying the process of humanness judgment and interaction dynamics in computer-mediated communication. Furthermore, they also provide new avenues for optimizing artificial agents designed to communicate with humans. Our results emphasize the collaborative aspect of dialogue, as well as the multi-dimensional and multi-factorial nature of this process. While classical descriptive analysis can provide important data, we also demonstrated that a complete analysis can not rely only on strictly descriptive factors, but should integrate cognitive and emotional dimensions in an integrated behavioral characterization. When creating synthetic agents, designers should keep in mind that equilibrium in the reciprocity of the exchanges between humans and agents is one of the central factors in order to convince a human about the humanness of its interlocutor. Finally, when taken together, our results strongly demonstrate that, in an interaction situation, the judgment of the humanness of an interlocutor not only depends of his behavior, but also on the judge himself.
